# Maternal nutrition altered embryonic *MYOD1*, *MYF5*, and *MYF6* gene expression in genetically fat and lean lines of chickens

**DOI:** 10.5713/ab.21.0521

**Published:** 2022-03-01

**Authors:** Feng Li, Chunxu Yang, Yingjie Xie, Xiang Gao, Yuanyuan Zhang, Hangyi Ning, Guangtao Liu, Zhihui Chen, Anshan Shan

**Affiliations:** 1 Institute of Animal Nutrition, Northeast Agricultural University, Harbin 150030, China

**Keywords:** Broiler Chicken, Embryo, Muscle Fibre, *MYOD1*, *MYF5*, *MYF6*

## Abstract

**Objective:**

The objectives of this study were to evaluate the effects of daily feed intake during the laying period on embryonic myogenic differentiation 1 (*MYOD1*), myogenic factor 5 (*MYF5*), and myogenic factor 6 (*MYF6*) gene expression in genetically fat and lean lines of chickens.

**Methods:**

An experiment in a 2×2 factorial design was conducted with two dietary intake levels (100% and 75% of nutrition recommendation) and two broiler chicken lines (fat and lean). Two lines of hens (n = 384 for each line) at 23th week of age were randomly divided into 4 treatments with 12 replicates of 16 birds. The experiment started at 27th week of age (5% egg rate) and ended at 54th week of age. Hatched eggs from the medium laying period were collected. Real time polymerase chain reaction analysis was used to analyse the *MYOD1*, *MYF5*, and *MYF6* mRNA levels of E7, E9, E11, E13, and E15 body tissues and E17, E19, and E21 chest and thigh muscle samples.

**Results:**

The results indicated that there were significant effects of line, dietary intake, and interactions between them on *MYOD1*, *MYF5*, and *MYF6* gene mRNA expression levels in embryonic tissues. Low daily feed intake did not change the expression trend of *MYOD1* mRNA in either line, but changed the peak values, especially in lean line. Low daily feed intake altered the trend in *MYF5* mRNA expression level in both lines and apparently delayed its onset. There was no apparent effect of low daily feed intake on the trends of *MYF6* mRNA expression levels in either line, but it significantly changed the values on many embryonic days.

**Conclusion:**

Maternal nutrient restriction affects myogenesis and is manifested in the expression of embryonic *MYOD1*, *MYF5*, and *MYF6* genes. Long term selection for fat deposition in broiler chickens changes the pattern and intensity of myogenesis.

## INTRODUCTION

Broiler chicken embryo development is a very complex process that ends in a successful emergence of the chick. When birds are born, the structure and function of skeletal muscle are usually mature [1]. Myogenic precursor cells called myoblasts are presumed to originate exclusively from mesoderm during vertebrate embryogenesis [2]. Muscle fibres are issued from myoblasts which proliferate, migrate, concentrate, fuse to multinucleated myotubes, and further differentiate into mature muscle fibers [3]. The activation of the myogenic programme and specification of muscle specific gene expression in the developing embryo is controlled by a family of 4 basic helix-loop-helix transcription factors, collectively known as the myogenic regulatory factors (MRFs), including myogenic differentiation 1 (*MYOD1*), myogenic factor 5 (*MYF5*), myogenic factor 6 (*MYF6*), and myogenin (*MYOG*) [4]. The four factors are expressed in a specific spatial and temporal sequence eventually allows the muscle to develop completely [5].

The effect of maternal nutrition on the muscles of offspring has focused on mammals, with few studies in poultry [6,7]. Many studies have demonstrated that egg composition and nutrition are affected by hens nutrition level [8,9]. As the only raw material and energy, the change of egg will inevitably affect myofiber formation [10]. Our previous studies showed that feed restriction could affect the expression of myostatin (*MSTN*) and *MYOG* genes [11]. We have reason to speculate that the expression of *MYOD1*, *MYF5*, and *MYF6* genes, as members of MRFs, will be affected and have both similar and unique responses between the two lines. In the present study, we examined the *MYOD1*, *MYF5*, and *MYF6* mRNA levels in chicken embryos on embryonic day (E) 7, E9, E11, E13, E15, E17, E19 and the day of hatching (E21) to evaluate the role of differential feed allocation on the muscle development of embryos during laying period in fat and lean lines.

## MATERIALS AND METHODS

### Animal care

The present experiment was reviewed and approved by the Northeast Agricultural University Laboratory Animal Ethics Committee (NEAUEC20090201).

### Birds and experimental design

The Northeast Agricultural University (NEAU) divergent broiler chicken lines for abdominal fat (lean line and fat line), which were selected by many generations through the rate of abdominal fat and the level of very low-density lipoprotein (VLDL) in plasma [12], were used. There was significant difference for abdominal fat weight and no difference for body weight between the lean and fat lines. Significant genetic differences between the two lines, especially in the control of abdominal fat content, were found in the previous study [12–15].

The experiment was a 2×2 factorial design of 2 factors. At 23rd week of age, 768 females (384 from each line) were randomly assigned into 4 treatments. There were 12 replicates in each group and 16 chickens in each replicate. The treatments were lean line and normal daily feed intake (LN), lean line and low daily feed intake (LL), fat line and normal daily feed intake (FN), fat line and low daily feed intake (FL). There was no significant difference in initial weight (means: 2,554.57 to 2,555.34 g; pooled standard error of the mean: 9.1341 g).

### Feeding and management

From 1 day to 24th week of age, all birds were given the same diet (0 to 6th week: 12.10 MJ ME/kg, 179 g/kg CP, 10.6 g/kg Ca; 7th to 18th week: 11.82 MJ ME/kg, 147 g/kg CP, 9.1 g/kg Ca; 19th to 24th week: 11.61 MJ ME/kg, 161 g/kg CP, 20.8 g/kg Ca). From the 24th week to 54th week, all the experimental groups were fed a standard maize-soy diet (11.80 MJ ME/kg, 170.9 g CP/kg, 9.2 g Lys/kg, 4.1 g Met/kg, 34.9 g Ca/kg, and 6.5 g P/kg). From 1 day to 26th week of age, all birds were fed the recommended daily feed intake (RDFI) of the Chinese Ministry of Agriculture (1 to 2 weeks: 15 g/bird/d; 3 to 4 weeks: 33 g/bird/d; 5 to 7 weeks: 41 g/bird/d; 7 to 8 weeks: 46 g/bird/d; 9 to 10 weeks: 51 g/bird/d; 11 to 12 weeks: 58 g/bird/d; 13 to 14 weeks: 66 g/bird/d; 15 to 16 weeks: 74 g/bird/d; 17 to 18 weeks: 84 g/bird/d; 19 to 20 weeks: 94 g/bird/d; 21 to 22 weeks: 107 g/bird/d; 23 to 24 weeks: 121 g/bird/d; 25 to 16 weeks: 136 g/bird/d) [16]. From 27th week to 54th week of age, hens in LN and FN treatments were fed the recommended RDFI (27 to 28 weeks: 150 g/bird/d; 29 to 32 weeks: 170 g/bird/d; 33 to 42 weeks: 167 g/bird/d; 43 to 57 weeks: 163 g/bird/d; 58-weeks 152 g/bird/d), and hens in LL and FL treatments were fed 75% of RDFI (27 to 28 weeks: 113 g/bird/d; 29 to 32 weeks: 128 g/bird/d; 33 to 42 weeks: 125 g/bird/d; 43 to 57 weeks: 122 g/bird/d; 58-weeks 114 g/bird). During laying period, hens were kept in a metal cage, with one replicate consisting of 16 hens in 8 adjoining cages. Replicates were equally distributed over the upper and lower cages to reduce cage space differences. Feed was given daily at 08:00 and 16:00 to reduce hunger and chronic stress. Water was freely available. The light was combined natural light and artificial light. During the early stages of the egg laying, the photoperiod gradually increased at 0.5 h weekly rate until 16 h per day. The temperature inside the house is controlled at about 20°C. The cage was disinfected through Potassium Permanganate and formalin.

### Sample collection

Fertile eggs by artificial insemination were obtained from each treatment replicate at 40th weeks and hatched in an automatic incubator (FT-ZF 10, Chunmingfangtong Electronic Co., LTD, Beijing, China). The body tissues from three embryos per replicate, without heads and internal organs, were taken from three eggs per replicate on E7, E9, E11, E13, and E15. Samples of pectoral muscle and thigh muscle were taken from three embryos per replicate on E17, E19 and E21. Tissue samples were quickly frozen in liquid nitrogen and kept at −80°C for real-time polymerase chain reaction (PCR) analysis.

### RNA extraction, reverse transcription (RT) and real-time polymerase chain reaction

According to the sequences of chicken *MYOD1* (NM_204214.1), *MYF5* (NM_001030363.1), *MYF6* (NM_001030746.1) and glyceraldehyde-3-phosphate dehydrogenase (GAPDH, NM_204305) published on the GenBank, oligonucleotide primer sets for the three genes were designed by Primer Premier 5.0 and Oligo 6.0 (Sangon Biological Engineering Technology & Services Co., Ltd, Shanghai, China). Parameters of gene-specific primers are described in Table 1. *GAPDH* were used as an internal standard gene.

Trizol Reagent Kit (Bioteke, Jiangsu, China) was used to isolate total RNA for RT-PCR analysis according to the manufacturer’s instructions. The purity of RNA was assessed spectrophotometrically by measuring absorbance at 260 and 280 nm (OD260/OD280: 1.8–2.0). DNA concentration was determined by measuring UV absorbance at 260 nm (U-0080D; Hitachi, Tokyo, Japan). The 1.2% agarose formaldehyde gel electrophoresis was used to assess RNA integrity [17].

A high-capacity cDNA reverse transcription kit (Applied Biosystems Inc., Foster City, CA, USA) was used to make all RNA samples converted into complementary DNA (cDNA). The specific operation process of cDNA reverse transcription was implemented according to the kit’s instruction manual [11].

Following the manufacturer’s instructions [11], the SYBR Prime Script TM RT-PCR Kit (DRR041A, Takara Bio Inc., Shiga, Japan) was used to amplify PCR. PCR amplification was performed in triplicate in an ABI PRISM 7500 Fast Real-time PCR system (Applied Biosystems, USA).

Quantification was accomplished according to the standard curve method as described by the PCR system manufacturer (Applied Biosystems, USA). In order to achieve the same PCR efficiency for each analyte, serial dilution of cDNA was used to construct standard curves for *MYOD1*, *MYF5*, *MYF6*, and *GAPDH* which was used as internal control. The R^2^ values for the standard curves of the test genes approached 1.0 suggesting the same amplification efficiency in the PCR reactions under these conditions. The expression levels of specific genes were normalized to the level of *GAPDH* expression in each sample. Fold-difference values were normalized relative to the average specific gene expression level in body tissues on E7.

### Statistical analysis

Experimental data were analysed by analysis of variance (ANOVA) using Proc Mixed of statistical analysis system (SAS Inst. Inc., Cary, NC, USA) for a randomized complete block with a factorial arrangement of treatments. The factorial treatment arrangement consisted of two dietary intake levels and two genotypes. Dietary intake and genotype were fixed; whereas blocks were random, the following model was used to analyse the data:


$$Y_{ijk}=\mu +\alpha_{i}+\beta_{j}+{\left(\alpha \beta \right)}_{ij}+P_{k}+{ɛ}_{ijk}$$

Where *Y_ijk_* = the value of individual samples, *μ* = overall mean, *α_i_* = dietary intake effect, *β_j_* = line effect, (*αβ*)*_ij_* = interaction between dietary intake and line, *P_k_* = effect of block, and *ɛ_ijk_* = error component. If differences in treatment means were detected by ANOVA, Duncan’s multiple range test was applied to separate means. A significance level of p<0.05 was used for analysis.

## RESULTS

### Detection of integrity for total RNA of embryonic tissue and real-time PCR products

**Figure 1:**
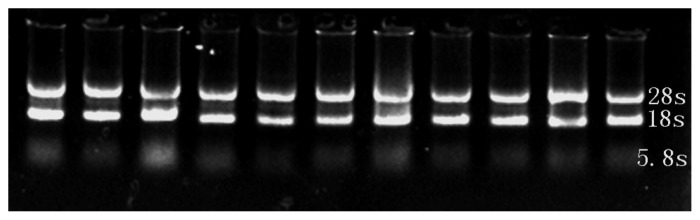
Detection of integrity for total RNA of embryonic tissue. RNA integrity was assessed by 1.2% agarose formaldehyde gel electrophoresis. The 18S and 28S ribosomal RNA bands are clearly visible in the RNA sample.

**Figure 2:**
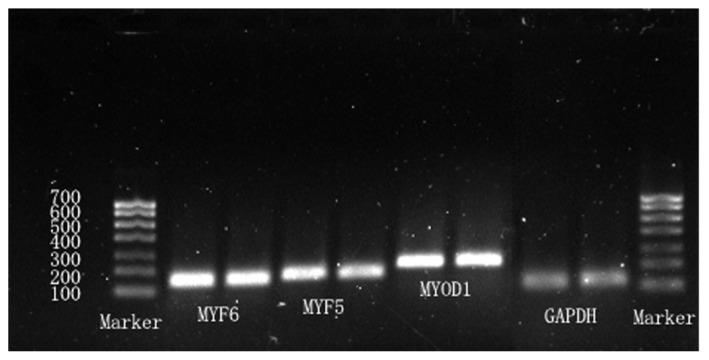
RT-PCR of *MYOD1*, *MYF5*, *MYF6*, and *GAPDH* mRNA of embryonic tissue. RT-PCR, reverse transcription-polymerase chain reaction; *MYOD1*, including myogenic differentiation 1; *MYF5*, myogenic factor 5; *MYF6*, myogenic factor 6; *GAPDH*, glyceraldehyde-3-phosphate dehydrogenase.

The intact total RNA of embryonic tissue was isolated and used as the initial sample to amplify *MYOD1*, *MYF5*, *MYF6*, and *GAPDH* genes by real-time PCR (Figure 1). cDNA fragments with sizes of 212, 167, 147, and 120 bps, respectively, were produced (Figure 2).

### The melting curve (dissociation curve), amplification curve and amplification efficiency curve (standard curve) of housekeeping gene and target genes

**Figure 3:**
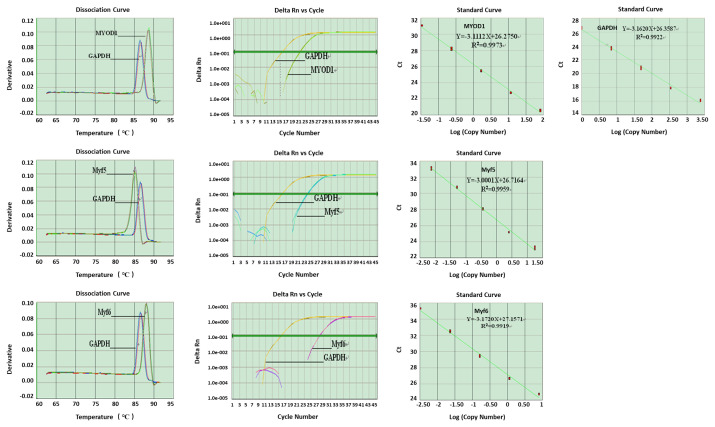
The dissociation, amplification and standard curves of housekeeping gene and target genes.

Housekeeping and target gene mRNA expression levels were surveyed by real-time PCR (Figure 3). There was only one specific peak in the dissociation curve of each gene, indicating that each one was specifically amplified with no primer dimer generated. The R^2^ values for all standard curves generated ranged between 0.99 and 1.0. The amplification efficiencies of the target genes were like that of the housekeeping gene.

### Relative expression levels of MYOD1, MYF5, and MYF6 mRNA in embryo tissues

Relative expression levels of *MYOD1* gene mRNA in embryonic tissues are presented in Table 2. Significant effects of and interactions between line and dietary intake were found for *MYOD1* gene mRNA expression levels of embryonic tissues. In both lines, low daily feed intake significantly decreased mRNA expression in body tissues on E13 and in thigh tissues on E21, and significantly increased mRNA expression in thigh tissues on E19. In the lean line, low daily feed intake significantly increased mRNA expression in body tissues on E9 and pectoral tissues on E19 but decreased in thigh tissues on E17. However, there were no effects observed in the fat line on those same days. In the fat line, low daily feed intake significantly increased mRNA expression in body tissues on E15 and then decreased in pectoral tissues on E17. However, there were no effects observed in the lean line on those days. The *MYOD1* gene mRNA expression levels of pectoral tissues on E17 and of thigh tissues on E21 were lower in the lean line than in the fat line.

Relative expression levels of *MYF5* gene mRNA in embryonic tissues are presented in Table 3. There were significant interactions between dietary intake and line on *MYF5* gene mRNA expression levels from E9 to E19. Low daily feed intake significantly increased *MYF5* mRNA expression levels in body tissues on E9 but significantly decreased the corresponding levels in the fat line. In the fat line, low daily feed intake significantly increased mRNA expression in body tissues on E7, in pectoral tissues on E17 and in thigh tissues on E19, and decreased expression in body tissues on E13, but there were no effects in the lean line on those days. In the lean line, low daily feed intake significantly increased mRNA expression in body tissues on E11 and E15 and decreased in thigh tissues on E17, but no effects were observed in the fat line on those days. In both lines, low daily feed intake significantly increased mRNA expression in pectoral tissues on E21. The *MYF5* gene mRNA expression levels of pectoral tissues on E17 and thigh tissues on E21 were higher in the fat line than in the lean line.

Relative expression levels of *MYF6* gene mRNA in embryonic tissues are presented in Table 4. Significant effects of and interactions between line and dietary intake were found for *MYF6* gene mRNA expression levels of embryonic tissues. Low daily feed intake significantly increased mRNA expression levels in body tissues of the lean line on E9 but significantly decreased the corresponding levels in the fat line. In the lean line, low daily feed intake significantly increased mRNA expression in thigh tissues on E19, but it decreased gene expression in pectoral tissues on E19 and E21. There were no effects observed in the fat line on those same days. In the fat line, low daily feed intake significantly decreased mRNA expression in body tissues on E13. However, there was no effect observed in the lean line on that day. In both lines, low daily feed intake significantly increased mRNA expression in body tissues on E7, in pectoral tissues on E17 and in thigh tissues on E21. The *MYF6* gene mRNA expression levels of body tissues on E13 were higher in the fat line than in the lean line, but those of body tissues on E15 and thigh tissues on E17 were lower conversely.

### Developmental changes of MYOD1, MYF5, and MYF6 mRNA expression

**Figure 4:**
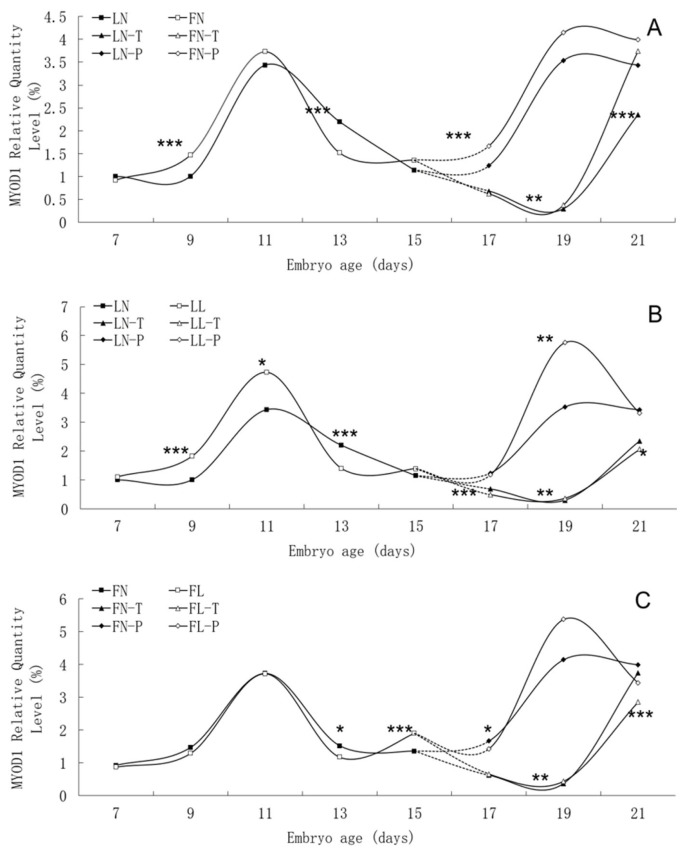
Developmental changes of *MYOD1* mRNA expression of embryos in genetically fat and lean lines of chickens. *MYOD1*, including myogenic differentiation 1; LN, body tissues in lean line with normal daily feed intake; LL, body tissues in lean line with low daily feed intake; FN, body tissues in fat line with normal daily feed intake; FL, body tissues in fat line with low daily feed intake; LN-T, thigh tissues in lean line with normal daily feed intake; LL-T, thigh tissues in lean line with low daily feed intake; FN-T, thigh tissues in fat line with normal daily feed intake; FL-T, thigh tissues in fat line with low daily feed intake; LN-P, pectoral tissues in lean line with normal daily feed intake; LL-P, pectoral tissues in lean line with low daily feed intake; FN-P, pectoral tissues in fat line with normal daily feed intake; FL-P, pectoral tissues in fat line with low daily feed intake. Stars indicate significant effects of line or daily feed intake. Levels of significance: * p<0.05, ** p<0.01, *** p<0.001.

There was similar trend in *MYOD1* mRNA expression level between the fat line and the lean line, although significant differences were found between them on many embryonic days, such as E9, E13, E17, E19 and E21 (Figure 4). In two lines, *MYOD1* mRNA expression in body tissues rose continuously from E7 to E11, peaked on E11, and then fell. There was different trend of *MYOD1* mRNA expression in pectoral tissues with that in thigh tissues from E17 to E21. There was no apparent effect of low daily feed intake on the trends of *MYOD1* mRNA expression levels in either line, but it significantly changed the values on many points, especially in lean line.

**Figure 5:**
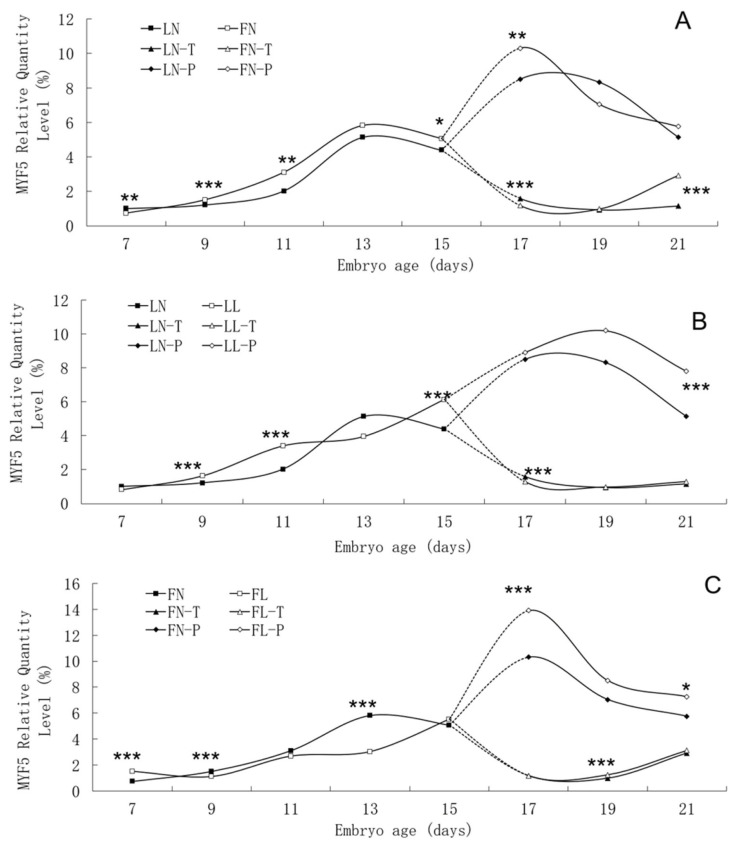
Developmental changes of *MYF5* mRNA expression of embryos in genetically fat and lean lines of chickens. *MYF5*, myogenic factor 5; LN, body tissues in lean line with normal daily feed intake; LL, body tissues in lean line with low daily feed intake; FN, body tissues in fat line with normal daily feed intake; FL, body tissues in fat line with low daily feed intake; LN-T, thigh tissues in lean line with normal daily feed intake; LL-T, thigh tissues in lean line with low daily feed intake; FN-T, thigh tissues in fat line with normal daily feed intake; FL-T, thigh tissues in fat line with low daily feed intake; LN-P, pectoral tissues in lean line with normal daily feed intake; LL-P, pectoral tissues in lean line with low daily feed intake; FN-P, pectoral tissues in fat line with normal daily feed intake; FL-P, pectoral tissues in fat line with low daily feed intake. Stars indicate significant effects of line or daily feed intake. Levels of significance: * p<0.05, ** p<0.01, *** p<0.001.

The trend in *MYF5* mRNA expression level during the embryonic period was similar between the two lines, although significant differences were found between them (Figure 5). In two lines, *MYF5* mRNA expression rose continuously from E7 to E13, peaked on E13, and then fell. A small increase in pectoral tissues occurred on E17. Low daily feed intake altered the trend in *MYF5* mRNA expression level in both lines, apparently delaying its onset.

**Figure 6:**
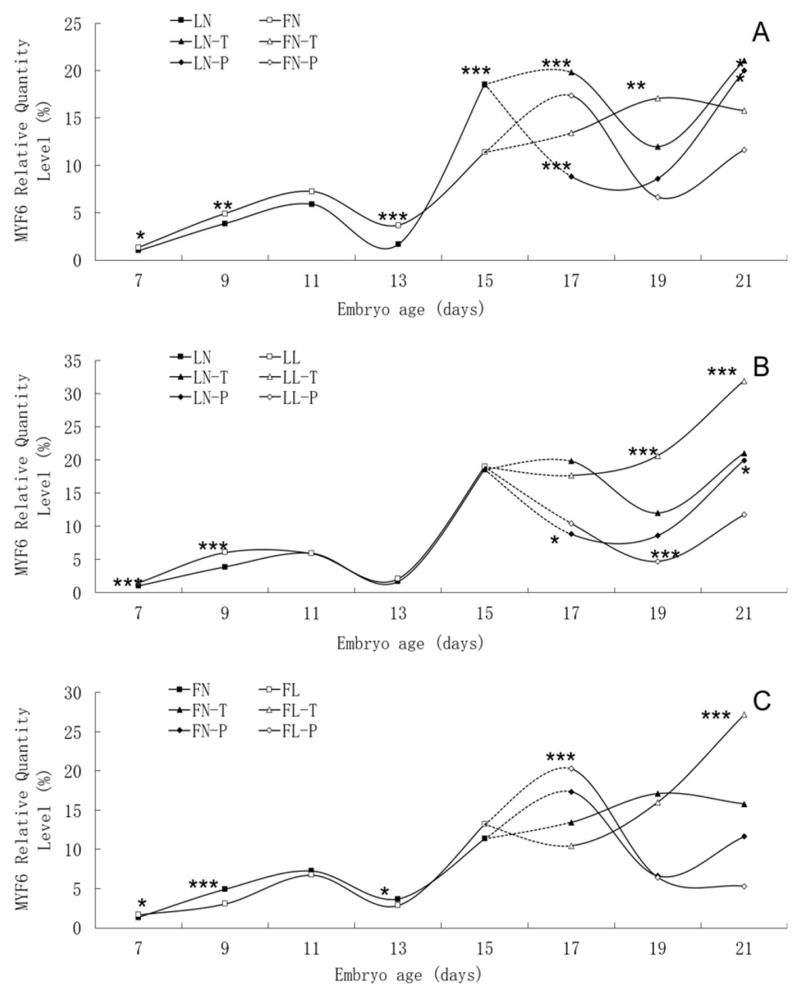
Developmental changes of *MYF6* mRNA expression of embryos in genetically fat and lean lines of chickens. *MYF6*, myogenic factor 6; LN, body tissues in lean line with normal daily feed intake; LL, body tissues in lean line with low daily feed intake; FN, body tissues in fat line with normal daily feed intake; FL, body tissues in fat line with low daily feed intake; LN-T, thigh tissues in lean line with normal daily feed intake; LL-T, thigh tissues in lean line with low daily feed intake; FN-T, thigh tissues in fat line with normal daily feed intake; FL-T, thigh tissues in fat line with low daily feed intake; LN-P: pectoral tissues in lean line with normal daily feed intake; LL-P, pectoral tissues in lean line with low daily feed intake; FN-P, pectoral tissues in fat line with normal daily feed intake; FL-P, pectoral tissues in fat line with low daily feed intake. Stars indicate significant effects of line or daily feed intake. Levels of significance: * p<0.05, ** p<0.01, *** p<0.001.

There was similar trend in *MYF6* mRNA expression level between the fat line and the lean line, although significant differences were found between them on many embryonic days, such as E7, E9, E13, E15, and E17 (Figure 6). In two lines, *MYF6* mRNA expression rose continuously from E7 to E11, peaked on E11, and then fell until to E13. High *MYF6* mRNA expression level was found in both lines from E15 to E21. There was no apparent effect of low daily feed intake on the trends of *MYF6* mRNA expression levels in either line, but it significantly changed the values on many points.

## DISCUSSION

In our experiment, the divergent broiler chicken lines for abdominal fat (lean line and fat line) were used, which have been selected divergently using abdominal fat percentage, plasma VLDL concentration and body weight as selection criteria by the team of Dr. Hui Li since 1996 [12]. Like our previous results about *MSTN* and *MYOG* [11], the present study showed that the overall trends of expression curves of *MYOD1*, *MYF5*, and *MYF6* were basically the same in two lines during embryonic development (from E7 to E21). These indicated that the selection for abdominal fat percentage, plasma VLDL concentration and body weight at 7th weeks of age did not affect the trend in *MYOD1*, *MYF5*, and *MYF6* mRNA expression over the embryonic period. The general process of myogenesis in different species (birds, rodents, mammals) is believed to be similar [3], and we suggested that there is a degree of programming in the expression of genes involved in muscle fibre development in two lines broiler chickens from the same breed. However, the significant differences in the *MYOD1*, *MYF5*, and *MYF6* gene mRNA expression level at multiple embryonic days between two lines showed long-term selection of fat deposition altered the strength of muscle embryonic development.

Feed restriction has been a routine practice in broiler breeder chicken management to reduce the body size to improve egg production [18]. However, several recent studies have shown that this practice causes alterations in the development of the progeny, both embryonic and post hatching [19,20]. These effects may be due to differences in the total amount of nutrients in eggs and maternal effects mediated by hormones, with the breeder under different nutritional stresses [21,22]. Similarly, although the present experiment specified a limit level of 25%, broiler breeder chickens of the two lines may have been subjected to different levels of energy deficit stress due to their different energy requirements. This was revealed in our other relevant studies on the effect of maternal restricted feeding on egg quality [8]. The lower nutrient allocation priority of skeletal muscle also makes it more vulnerable to nutrient deficiency [23]. The expression of *MSTN* and *MYOG* in response to maternal restricted feeding at laying stage has been illustrated in our previous study [11].

*MYOD1* and *MYF5* are expressed in proliferating myoblasts and play early roles in the determination of muscle precursor cells to the myogenic lineage [24]. *MYF5* and *MYOD1* have similar and overlapping functions during myoblast determination [25,26]. The function of *MYOD1* during embryonic development is to connect mesoderm cells to a skeletal myoblast lineage, and then to constantly adjust that activity [27]. Evidence also suggests *MYF5* functions more towards proliferation whilst *MYOD1* prepares myoblasts for efficient differentiation [28]. During the 3-week period of chick embryonic development, myogenesis occurs in two waves, sequentially generating two populations of myotubes, which are the primary myotubes formed between E4 and E7 and the secondary myotubes formed between E8 and E16 [29]. One study showed that more than 95% of myoblasts began to proliferate at the time of secondary myogenesis [30]. In the present experiment we observed the peak expression of *MYOD1* at E11. However, no clear study has shown a positive relationship between *MYOD1* expression level and the and myoblast proliferation rate [5].

Furthermore, unlike the primary muscle fibres regulated mainly genetically, the number of secondary fibres will be affected by maternal nutrition [31]. In the lean line, *MYOD1* and *MYF5* is expressed rapidly in large quantities from E9 to E11s, which may mean that myoblasts from lean line embryos proliferate earlier and prepare for subsequent differentiation. For fat line, the *MYOD1* and *MYF5* gene mRNA expression levels decreased at E13 due to feed restriction. We speculate that this is a result of the different energy pressures exerted by the two lines, with the fat line being under stronger pressure. The results of expression changes of *MYOG* [11], as a factor regulating myoblast differentiation and to some extent representing the number of myofibers, similarly support this conclusion. The peak value of *MYOD1* gene expression in pectoral tissues on E19 was increased significantly by low maternal daily feed intake in the lean line. And in the fat line, a peak value of *MYF5* gene mRNA expression in pectoral tissues on E17 appeared and was increased significantly by low maternal daily feed intake. *MYOD1* and *MYF5* are likely to function in two distinct cell lineages [32]. If the reason here is that restricted feeding of the mother decreases the number of muscle fibres in broiler chickens. The decrease in different genes may be due to the myofiber developmental lineage of both lines being altered by long-term breeding for fat deposition, which is worth further exploration.

*MYF6* also known as myogenic regulatory factor 4 (*MRF4*) and herculin is expressed transiently during the early stages of myoblast proliferation, but is mainly expressed in postnatal skeletal muscle tissue where it is supposed to be important for maintenance of skeletal muscle fibre differentiation-specific phenotype [5]. Many researches indicated *MYF6* was expressed mainly after birth [33,34]. In this study, *MYF6* mRNA expression level was very low in the middle stage and increased in the late stage of embryonic development, which was consistent with former researches. Moreover, unlike *MYOD1* and *MYF5*, *MYF6* is also expressed at higher levels in thigh muscles during late embryogenesis to the same level as in pectoral muscles. This is related to its function in maintaining muscle fibre types. Besides that, maternal dietary intake restriction had an opposite effect on *MYF6* gene mRNA expression in the pectoral and thigh tissues of both lines on E17–E21. This may be caused by the different maturation times of the pectoral and thigh muscles.

However, given the complexity of muscle embryonic de velopment, our discussion of the impact of this difference is not comprehensive. These results indicated *MYF6* gene mRNA expression could be affected by maternal nutrition. Subsets of undifferentiated muscle precursor cells, namely the satellite cells, appear during late chicken embryogenesis between 13 and 16 days in ovo [35,36]. The satellite cells remain associated with the developing muscle fibers and play prominent roles in adult muscle regeneration and growth [37]. In another study focused on the effect of maternal nutrition on meat quality of broilers, compensatory muscle growth was found in the offspring of restricted feeding treatment [38]. During the last few days of embryonic development, muscle fibers begin to differentiate into different fiber types [39]. Unfortunately, we do not know how maternally restricted feeding in this study affected the satellite cell and muscle typing processes and whether this effect would be manifested in the expression of MRFs genes. The mRNA and protein expression of *MYOD1* is abundantly expressed in fast muscle fiber in vertebrates [40,41]. And in this study, *MYOD1* and *MYF5* were expressed at higher levels in breast tissues than in thigh tissues from E17 to E21. In addition, if the remaining yolk sac is not sufficient for the full nutritional requirements of the hatching activity of very mature avian embryos, a situation in which atrophy occurs in the production of pectoral muscles with predominantly type IIB fibers [42].

## CONCLUSION

Selection for abdominal fat percentage, plasma VLDL concentration and body weight at 7th week of age affected the embryonic myogenesis by significantly changing *MYOD1*, *MYF5*, and *MYF6* gene mRNA expression levels in embryonic tissues. The embryonic myogenesis was also affected by maternal daily feed intake. Low maternal daily feed intake significantly changed the peak values of *MYOD1*, *MYF5*, and *MYF6* mRNA expression in chicken embryos. long-term selection of fat deposition altered the response of the expression of *MYOD1*, *MYF5*, and *MYF6* genes to the interference of maternal nutrition.

## Figures and Tables

**Table 1:** Parameters of gene-specific primers for *MYOD1*, *MYF5*, *MYF6*, and *GAPDH* genes

Target genes	GenBank accession number	Primer sequence (5′→3′)	Position	Product size (bp)	Annealing temperature (°C)
*GAPDH*	NM_204305	PF: 5′-GCCATCACAGCCACACAGA -3′	589	120	57.7
		PR: 5′-TTTCCCCACAGCCTTAGCA -3′	708		
*MYOD1*	NM_204214.1	PF: 5′-AACGCCATCCGCTACATC-3′	427	212	57.7
		PR: 5′-TCATTTGGTGATTCCGTGTAG-3′	638		
*MYF5*	NM_001030363.1	PF: 5′-GGCTGAAGAAAGTGAACCAAGT-3′	290	167	56.4
		PR: 5′-TCCCGGCAGGTGATAGTAGTT-3′	456		
*MYF6*	NM_001030746.1	PF: 5′-GGAGCGCCATCAGCTACATC-3′	404	147	59.6
		PR: 5′-CGCAGGTGCTCAGGAAGTCT-3′	550		

*MYOD1*, myogenic differentiation 1; *MYF5*, myogenic factor 5; *MYF6*, myogenic factor 6; *GAPDH*, glyceraldehyde-3-phosphate dehydrogenase.

**Table 2:** Effect of maternal dietary intake on the fold difference of *MYOD1* gene mRNA in embryonic tissues of lean line and fat line hens (n = 36 per treatment)

Embryo age (d)	Group^1)^				Pooled SEM	Probability values		
	
	LN	LL	FN	FL		Line	Feed intake	Line×intake
7	1.000^ab^	1.099^a^	0.919^b^	0.872^b^	0.0799	0.007	NS	NS
9	1.003^c^	1.813^a^	1.464^b^	1.290^b^	0.1095	NS	<0.001	<0.001
11	3.434	4.725	3.732	3.718	0.6331	NS	NS	NS
13	2.199^a^	1.391^bc^	1.516^b^	1.174^c^	0.1308	<0.001	<0.001	0.013
15	1.140^b^	1.383^b^	1.354^b^	1.902^a^	0.1384	<0.001	<0.001	NS
17 (Thigh)	0.681^a^	0.488^b^	0.623^a^	0.661^a^	0.0462	NS	0.019	<0.001
17 (Pectoral)	1.236^bc^	1.177^c^	1.668^a^	1.433^b^	0.1000	<0.001	0.040	NS
19 (Thigh)	0.293^c^	0.363^b^	0.372^b^	0.441^a^	0.0237	<0.001	<0.001	NS
19 (Pectoral)	3.534^c^	5.757^a^	4.144^bc^	5.372^ab^	0.7352	NS	0.001	NS
21 (Thigh)	2.351^c^	2.063^d^	3.748^a^	2.858^b^	0.1340	<0.001	<0.001	0.002
21 (Pectoral)	3.435	3.307	3.992	3.439	0.4625	NS	NS	NS

*MYOD1*, myogenic differentiation 1; SEM, standard error of the mean; NS, non significant.

1) LN, lean line and normal daily feed intake; LL, lean line and low daily feed intake; FN, fat line and normal daily feed intake; FL, fat line and low daily intake.

a–c Means within a row with no common superscripts differ significantly (p<0.05).

**Table 3:** Effect of maternal dietary intake on the fold difference of *MYF5* gene mRNA in embryonic tissues of lean line and fat line hens (n = 36 per treatment)

Embryo age (d)	Group^1)^				Pooled SEM	Probability values		
	
	LN	LL	FN	FL		Line	Feed intake	Line×intake
7	1.000^b^	0.823^bc^	0.735^c^	1.513^a^	0.0901	0.001	<0.001	<0.001
9	1.211^c^	1.634^a^	1.507^b^	1.116^c^	0.0626	0.014	NS	<0.001
11	2.024^c^	3.400^a^	3.103^ab^	2.686^b^	0.3316	NS	0.043	<0.001
13	5.139^ab^	3.951^bc^	5.820^a^	3.036^c^	0.6277	NS	<0.001	0.074
15	4.391^c^	6.129^a^	5.070^b^	5.537^b^	0.2772	NS	<0.001	0.002
17 (Thigh)	1.587^a^	1.285^b^	1.176^b^	1.181^b^	0.0820	<0.001	0.011	0.009
17 (Pectoral)	8.505^c^	8.904^c^	10.311^b^	13.910^a^	0.5549	<0.001	<0.001	<0.001
19 (Thigh)	0.940^b^	0.962^b^	0.979^b^	1.248^a^	0.0679	0.001	0.003	0.011
19 (Pectoral)	8.321^ab^	10.199^a^	7.044^b^	8.523^ab^	1.0952	NS	0.033	NS
21 (Thigh)	1.154^b^	1.287^b^	2.922^a^	3.123^a^	0.1682	<0.001	0.163	NS
21 (Pectoral)	5.151^b^	7.815^a^	5.758^b^	7.270^a^	0.6121	NS	<0.001	NS

*MYF5*, myogenic factor 5; SEM, standard error of the mean; NS, non significant.

1) LN, lean line and normal daily feed intake; LL, lean line and low daily feed intake; FN, fat line and normal daily feed intake; FL, fat line and low daily intake.

a–c Means within a row with no common superscripts differ significantly (p<0.05).

**Table 4:** Effect of maternal dietary intake on the fold difference of *MYF6* gene mRNA in embryonic tissues of lean line and fat line hens (n = 36 per treatment)

Embryo age (d)	Group^1)^				Pooled SEM	Probability values		
	
	LN	LL	FN	FL		Line	Feed intake	Line×intake
7	1.000^c^	1.505^ab^	1.339^b^	1.667^a^	0.1463	0.016	<0.001	NS
9	3.866^c^	6.062^a^	4.920^b^	3.071^d^	0.3159	<0.001	NS	<0.001
11	5.911	5.908	7.256	6.739	1.2520	NS	NS	NS
13	1.650^c^	2.096^c^	3.675^a^	2.876^b^	0.3716	<0.001	NS	0.019
15	18.565^a^	19.022^a^	11.373^b^	13.210^b^	1.5446	<0.001	NS	NS
17 (Thigh)	19.838^a^	17.664^a^	13.440^b^	10.466^b^	1.6236	<0.001	0.027	NS
17 (Pectoral)	8.846^d^	10.469^c^	17.363^b^	20.309^a^	0.6992	<0.001	<0.001	NS
19 (Thigh)	11.987^c^	20.648^a^	17.092^b^	15.996^b^	1.5446	NS	0.009	<0.001
19 (Pectoral)	8.604^a^	4.695^b^	6.660^ab^	6.446^b^	1.0871	NS	0.009	0.018
21 (Thigh)	21.036^c^	31.980^a^	15.797^d^	27.151^b^	2.3414	0.003	<0.001	NS
21 (Pectoral)	19.977^a^	11.734^b^	11.637^b^	5.286^b^	3.7559	0.006	0.007	NS

*MYF6*, myogenic factor 6; SEM, standard error of the mean; NS, non significant.

1) LN, lean line and normal daily feed intake; LL, lean line and low daily feed intake; FN, fat line and normal daily feed intake; FL, fat line and low daily intake.

a–d Means within a row with no common superscripts differ significantly (p<0.05).
